# Evaluation of the relationship between lithium treatment response and suicide attempt in bipolar disorder

**DOI:** 10.1192/j.eurpsy.2022.1031

**Published:** 2022-09-01

**Authors:** K. Altınbaş

**Affiliations:** Selçuk University, Psychiatry, Konya, Turkey

**Keywords:** suicid, alda scale, bipolar disorder, lithium treatment

## Abstract

**Introduction:**

Suicide risk is 20-30 fold higher in bipolar disorder(BD) than general population. A positive family history of suicide, early-onset bipolar disorder, rapid cycling, and drug/alcohol addiction have been identified as risk factors for non-fatal suicidal behavior. Lithium is the only mood stabilizer known to have a suicide-reducing effect in patients with BD. Less than half of the bipolar patients respond lithium well. Even though mechanism of action on suicide behavior is not clearly known, it is thought that lithium significantly reduces “impulsive-aggressive” behavior via serotonergic system which might also be related with treatment response in BD.

**Objectives:**

The aim of this study is to evaluate the relationship between lithium response and history of suicide.

**Methods:**

Those who scored 7 points or more from the Alda total score were considered good responders. Patients were divided into those who responded well to lithium treatment and those who did not. History of suicide attemptbetween these two groups was compared.

**Results:**

65.3% of the patients were female (n:49). The mean age of the patients was 36.82±13.35 years. 25 patients responded well to lithium treatment. Among the good responders, 32% of the patients and 25% of the non-responders had a history of suicide attempts. This difference was not statistically significant. (p=0.46 x²=0.13)

**Conclusions:**

The insufficient number of data in the study was considered as a limitation of this study. In addition, there is a need for more studies as there are many factors that cause suicide attempts.

**Disclosure:**

No significant relationships.

